# Anticancer Potential and Capsianosides Identification in Lipophilic Fraction of Sweet Pepper (*Capsicum annuum* L.)

**DOI:** 10.3390/molecules25133097

**Published:** 2020-07-07

**Authors:** Barbara Chilczuk, Beata Marciniak, Anna Stochmal, Łukasz Pecio, Renata Kontek, Izabella Jackowska, Małgorzata Materska

**Affiliations:** 1 Department of Chemistry, Faculty of Food Science and Biotechnology, University of Life Sciences in Lublin, Akademicka 15, 20-950 Lublin, Poland; barbara.chilczuk@up.lublin.pl (B.C.); izabella.jackowska@up.lublin.pl (I.J.); 2 Department of Molecular Biotechnology and Genetics, Faculty of Biology and Environmental Protection, University of Lodz, Banacha 12/16, 90-237 Lodz, Poland; beata.marciniak@biol.uni.lodz.pl (B.M.); renata.kontek@biol.uni.lodz.pl (R.K.); 3 Department of Biochemistry and Crop Quality, Institute of Soil Science and Plant Cultivation, State Research Institute, Czartoryskich 8, 24-100 Puławy, Poland; asf@iung.pulawy.pl (A.S.); lpecio@iung.pulawy.pl (Ł.P.)

**Keywords:** *Capsicum annuum*, phenolic compounds, antiradical activity, anticancer properties, capsianoside, mass spectrometry, ^1^H- and ^13^C-NMR

## Abstract

This study aimed to determine the health-promoting properties of sweet pepper by comparing the activity of fractions with variable lipophilicity. Fractions from red pericarp: aqueous (F1), 40% MeOH (F2), and 70% MeOH (F3) were analyzed for antiradical activity (with DPPH^•^ and ABTS^+•^), and the contents of total phenolic compounds (TP), flavonoids (TF), and dihydroxycinnamic acids (TDHCA). The anticancer potential of the fractions was evaluated in vitro using different cancer cell lines: human colorectal carcinoma (HCT116) and PC-3 (prostate cancer cell). Fibroblast-like cells of L929 obtained from subcutaneous adipose tissue of mouse were used as normal cells. The highest content of TP, TF, and TDHCA along with the strongest antiradical activity was observed for fraction F2, while the strongest anticancer properties against PC-3 were observed in fraction F3. Fraction F3 primarily contained capsianoside derivatives, which had been isolated through chromatographic methods and identified by spectral methods. These analyses helped in identifying 8 compounds, including 3 new compounds.

## 1. Introduction

Owing to the rapid increase in lifestyle-related diseases, public health has become a key element in the existence of societies and quality of life in general. Health-oriented food is now being recognized as an important factor that can prevent these diseases, especially foods of natural origin [1,2]. Natural food can be a rich source of compounds with health-promoting properties, which are characterized by a milder, yet more complex effect on the body as compared to their synthetic counterparts [3]. Often, the reconstitution of these compounds through chemical synthesis is impossible owing to their complex structure. Therefore, plants remain the source of many active substances, and research focused on their activity is growing in the last decade [2]. Capsaicinoids present in peppers (*Capsicum*) [4], rosemary ethanol extracts (*Rosmarinus officinalis* L.) [5], and *Berberis cretica* extracts [6] or substances contained in ginger [7] are some examples that have been studied so far.

Many of the plant-derived compounds have been known for their pharmacological activities [8]. Some of them are typical antioxidants like vitamin C or E [9,10], while others are plant pigments like carotenoids and flavonoids [11]. Phenolic acids also form an important group, which include cinnamic acid derivatives: chlorogenic and ferulic. They demonstrate the ability to inhibit the development of tumors and the formation of mutagenic compounds, e.g., nitrosamines [12,13]. It has been found that dietary polyphenols are capable of crossing the blood-brain barrier and can successfully treat central nervous system diseases [14]. For example, ferulic and caffeic acids have been found to delay the development of Alzheimer′s disease [15,16]. Chlorogenic acid plays an important role in the chemoprevention of neoplastic diseases because it has anti-inflammatory properties and inhibits the transformation of γ-aminobutyric acid (GABA) in the central nervous system [12].

The subject of the study was the sweet red pepper fruit, hugely popular and widely available all over the world. The extracts obtained from red pepper can be used as an alternative to artificial colors and preservatives [4]. Pepper fruits are rich in antioxidants (vitamins C, E, and β-carotene), as well as bioflavonoids [9,17]. The substances that provide the pungent property to pepper are a group of lipophilic alkaloids, the capsaicinoids which are amides of nonenoic acid and vanillin [18,19]. The main capsaicinoid is capsaicin, which is the pungent component in chili pepper, and it is known to have analgesic properties and anti-obesity effects [2,20]. However, studies indicate that capsaicin may have both beneficial and harmful effects on health [19,21], sometimes acting as antitumor agents and other times as carcinogens [21,22]. Capsaicin have analogs which occur in sweet pepper, those are capsinoids, and the primary natural capsinoid is capsiate [23]. The chemical structures of capsinoids are similar to those of the main capsaicinoids, except for the center linkage, which is an ester bond of vanillyl alcohol in capsinoids and an amide bond in capsaicinoids [17,24]. Just like capsaicinoids, capsinoids have also attracted great attention due to their extensive pharmacological properties [21,25,26,27,28,29,30]. In comparison to capsaicin, capsiate has less pungent irritancy, which facilitates daily intake. Several studies have revealed that capsinoids raise energy expenditure and suppress body fat accumulation in mice [23]. Other studies indicated similar or greater biological activity of capsinoids than capsaicinoids, such as improving insulin sensitivity, lipid, and glucose metabolism [27]. A rarely studied group of compounds found in sweet pepper are capsianosides, which are diterpenoid glycosides present in the lipophilic fraction of pepper constituents.

For this reason the present study aimed to compare the activity of fractions obtained from the ethanolic extract of sweet pepper fruit with variable lipophilicity and select the fraction with the strongest biological abilities measured by antioxidant activity and anticancer potential. Furthermore, the qualitative composition of the fraction displaying the most potent anticancer properties was evaluated, and the structures of the compounds that determine anticancer properties were elucidated.

## 2. Results and Discussion

### 2.1. Phenolic Content

Table 1 presents the results of the quality of the ethanol extract (E), water (F1), methanol-water 40% (F2), and 70% (F3) fractions obtained from the sweet pepper fruit, manifested as total phenolics (TP), total flavonoids (TF), and total dihydroxycinnamic acids (TDHCA). These fractions contained significant amounts of extractable compounds. Variability in the extracted yields is ascribed to the difference in lipophilicity of compounds present in the fruits. The maximum value of total extractable compounds was found in the ethanol extract (E), and then in the water fraction (F1), whereas the lowest values were observed for methanol-water 40% (F2) (Table 1). This shows that compounds extracted with an 80% ethanol solution have a stronger affinity to water than to lipophilic solvents. Hence, they were eluted in the aqueous fraction during SPE.

For TP, the highest level was characterized by the fraction F2 and the lowest by the ethanol extract (E). The F2 fraction also contained the highest concentration of TF and TDHCA. The order of TP content obtained for the three fractions and ethanolic extract of sweet pepper fruit was found to be F2 > F3 > F1 > E, for TF was F2 > E ≥ F1 > F3 and for TDHCA was F2 > F3 > E ≥ F1 (Table 1). The results acquired in these experiments are confirmed by previous studies in 40% fraction of methanol-water, which was rich in phenolic compounds, especially in flavonoid glycosides, and glycosidic derivatives of ferulic and sinapic acid which have been identified in different varieties of sweet pepper fruits [17]. The Folin-Ciocalteau method for TP analysis is associated with the oxidizing potential of the extracts [31,32]. These relationships are validated by tests of antiradical activity.

### 2.2. Antiradical Activity

Methods based on antiradical activity measurements are frequently used to assess the quality of natural raw materials and food. A distinctly common measurement system is the reaction with the synthetic DPPH^•^ radical, which is most frequently used to evaluate the antioxidant properties of phenolic compounds [32]. One of the disadvantages of this method is the fact that DPPH^•^ dissolves only in organic solvents and does not allow the determination of hydrophilic antioxidants [33,34]. Hence, another redox model system should be used alongside the DPPH radical, which will include measurements of the activity of hydrophilic compounds. The ABTS^+•^ cation radical method enables the determination of the antioxidant capacity of samples with both hydrophilic and lipophilic properties. Antiradical activity tests of the obtained fractions validated the dependence of the antioxidant property on the composition of the fraction with variable lipophilicity. Fraction F2, with the highest level of TP, TF, and TDHCA showed the highest level of activity toward DPPH^•^ and ABTS^+•^ than the others (Table 1). These results are in line with the results of previous studies, where the high correlation between antioxidant activity measured against DPPH^+•^ and ABTS^•^ radicals with flavonoids and cinnamic acid content was demonstrated [31,32,35]. The existing research data prove that phenolic compounds, especially flavonoids that exhibit antioxidant properties in a hydrophilic system need not exhibit them in a lipophilic medium [36]. These results were validated in this work for fraction F2, where a 50% reduction of ABTS^+•^ was noted in a four-fold lower fraction concentration as for DPPH^•^ (Table 1). The scavenging tests gave negative results for fraction F3. These results are at par with those obtained by Marino et al. [37], who noted that acyclic diterpenes named capsianosides isolated from sweet pepper fruits were inactive with relation to DPPH radicals as well as in reduction Cu^2+^/Cu^+^ and lipid peroxidation tests.

### 2.3. Biological Activity

The tested fractions showed a different relationship between anticancer activity and the content of phenolic compounds and antioxidant activity (Table 1). The most promising results were obtained for low in phenols F3 fraction. It has exhibited significant cytotoxicity against tested cancer cells (IC_50_ = 51 µg/mL) and it simultaneously was not severely toxic for normal fibroblasts L929 (IC_50_ = 94 µg/mL). The similar IC_50_ value = 60 µg/mL was obtained after treatment of PC-3 cells with the F1 fraction. Although, the all tested fractions were less cytotoxic for normal fibroblasts L929 than for PC-3 cancer cells, the highest IC_50_ values were obtained for HCT116 cells. Thereby, tested fractions (F1–F3) demonstrated a carcinoma-specific cytotoxicity against human prostate cancer cells, but in contrast, less cytotoxic for colon carcinoma cells HCT116. The observed differences in the cytotoxic activity of the F3 fraction between HCT116 and PC3 tumour cell lines may be due to the fact, that different types of tumours show different levels of sensitivity due to variable characteristics such as unique gene expression profiles. For example PC-3 harbours a mutation in *TP53* gene or the null mutation exist while, HCT116 possess a wild type variant of the gene. The effectiveness of chemical compounds is related also to the dynamics of cancer development, duration of cell cycle in a given type of cancer (the doubling time for HCT116 17.4 h vs. 27.1 h for PC-3). These parameters are different for particular types of cancers and can affect the response of individual cancer cell lines to tested compounds or group of compounds.

The studies presented in the publication belong to the screening tests. On the basis of the conducted research, further experiments will be done to find out the mechanisms of action of the tested fractions and individual chemical compounds present in the previously mentioned fractions. The obtained correlation between normal fibroblasts (lower cytotoxicity) and PC-3 cells (higher cytotoxicity) is one of the positive effects in the cytotoxicity studies of new groups of compounds. Lipophilic compounds present in sweet pepper fruits—Capsicum annuum L. are mainly derivatives of capsinoids that show biological properties similar to their acute analogs—capsaicinoids [19]. In some cases, nonpungent capsiate shows more pro-health properties than capsaicinoids. It was observed that capsiate enhanced glucose homeostasis and manifested higher antidiabetic activity than capsaicin [21]. This, in turn, enhances lipid and glucose metabolism, and, in this manner, the capsiate exhibits anti-obesity effects in animals and humans [38]. For anticancer activity, only limited information regarding the activity of capsinoids is available in the literature. Macho et al. [28] showed that capsiates pick out a cellular redox system and successfully prevent tumorogenesis in athymic mice. Also, Pyun et al. [39] proved that capsiate and dihydrocapsiate retained anticancer and chemopreventive activity similar to capsaicin, but without the pungent property. With regard to capsianosides present in sweet pepper, there is limited data on the anticancer properties of these compounds. Nevertheless, the antihypertensive and antibacterial properties of those compounds were noted by Marino et al. [37]. To study the qualitative composition of the F3 fraction, a preparative separation of its components was performed.

### 2.4. Isolation and Identification

**Figure 1:**
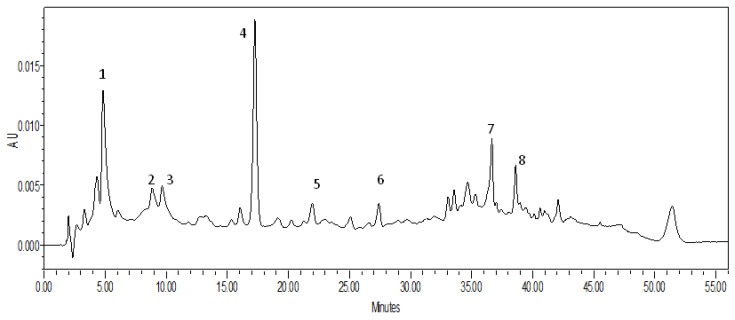
Chromatogram of the lipophilic fraction (F3) obtained from sweet pepper fruit. Numbered peaks are listed in Table 2.

Due to the preparative separation of the lipophilic fraction from sweet pepper fruits and combined spectral analytical methods, 8 compounds belonging to the group of diterpenic glycosides were isolated and identified (Table 2). Figure 1 presents the chromatogram of the F3 fraction along with marked numbers of determined compounds.

**Figure 2:**
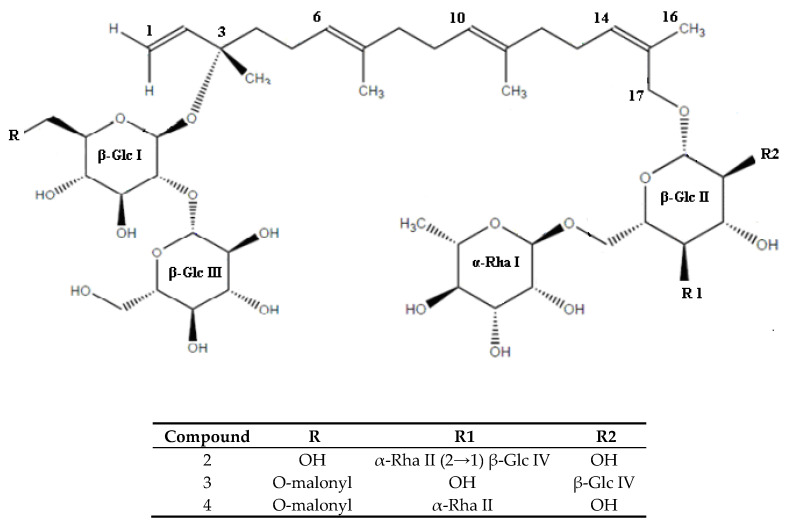
Structural representation of the newly identified compounds.

The qualitative analysis of pure compounds was based on ^1^H-NMR and ^13^C-NMR spectroscopic examination and ESI-MS mass spectrometry. The identification of the compounds was based on comparisons of the results obtained with data available in the literature. Five of the identified substances were known compounds: Capsianoside I, III, IV, VIII, and IX (Table 2) [37,40,41]. Three compounds, numbered **2, 3**, and **4**, have not yet been described in the literature. Table 3 presents the results of ^1^H-NMR and ^13^C-NMR spectroscopic analysis of these compounds. The comparative analysis of ^1^H-NMR, ^13^C-NMR, and ^1^H-^1^H spectroscopic correlation spectrum shows that the identified new compounds also belong to the group of acyclic diterpenic glycosides, in which the basic framework was 6*E*,10*E*,14*Z*,17-hydroxygeranyllinalool. The differences between the structure of the identified compounds were seen in their oligosaccharide chains (Figure 2).

In compound **2**, with the molecular formula C_56_H_94_O_30_, six sugar units were present (Figure 2) which was confirmed by proton resonances at δ_H_ 4.47, 4.56, 4.20, 4.58, 4.71, and 4.82, which were correlated in the HSQC spectrum with their equivalent carbon signals at δ_C_ 98.4, 105.9, 102.1, 105.6, 101.6, and 102.3 ppm. The ^13^C-NMR spectrum showed the presence of 17 carbon atoms in the base chain including one oxidized methylene carbon (C-17, 67.7 ppm), one oxygenated tertiary carbon (C-3, 82.1 ppm) along with four β-glucose signals and two α-rhamnose signals. Comparative NMR analyses suggested the presence of the same disaccharide chain as in the molecule of Capsianoside VIII. The HMBC and selective 1D TOCSY experiments established that this chain consists of 2 glucose molecules GLC III and GLC I (C-2). The remaining sugar units—glucose × 2 and rhamnose × 2 were components of the second oligosaccharide chain in which the glucose unit Glc II was combined with C-17 aglycone, Rha I (δ_H_ 4.71) and merged with Glc II at position C-6 (δ_C_ 66.8). In the same analysis, correlations of H-1 Rha II (δ_H_ 4.82) with Glc II in position C-4 (δ_C_ 78.9) were observed. The last sugar of the described chain was Glc IV, which merged with Rha II in position C-4 (δ_C_ 83.3) (Figure 2). The glucose configuration is assumed to take the D form, which is compatible with the stereochemistry of the naturally occurring isomer [42].

Therefore, compound **2** is 3-*O*-β-d-glucopyranosyl-(1→2)-β-d-glucopyranosyl-17-hydroxy-6*E*,10*E*,14*Z*-(3*S*)-geranyllionalool-17-*O*-[{β-d-glucopyranosyl-(1→2)}α-l-rhamnopyranosyl-(1→4)-{α-l-rhamnopyranosyl-(1→6)}]-β-d-glucopyranoside (Table 3, Figure 2).

Like compound **2**, compound **3** with the molecular formula C_53_H_86_O_29_ also included the main carbon chain formed with 17 carbon atoms and two oligosaccharide chains (glycosylated in positions C-3, C-17) (Figure 2). The first one contained two glucose molecules correlated in the position Glc I C2 (82.9)-Glc III C1 (105.8). The presence of a carbon chain consisting of 3 carbon atoms was observed in the position C6 (δ_C_ 65.6 ppm) Glc I, where C-1 and C-3 (168.5, 170.1ppm) were secondary carbon atoms linked with oxygen atoms. The second oligosaccharide chain (C-17) consisted of 3 sugar molecules: Glc II, Glc IV, and Rha I. The study showed that it was a 2,6 diglycosylated D-glucopyranosyl unit. Rha I (δ_H_ 4.75) correlated with Glc II in position C-6 (δ_C_ 67.6) and Glc IV (δ_H_ 4.63) with Glc II in position C-2 (δ_C_ 81.9). Thus, compound **3** is 3-*O*-β-d-glucopyranosyl-(1→2)-β-d-glucopyranosyl-6-malonyl-17-hydroxy-6*E*,10*E*,14*Z*-(3*S*)-geranyl-linalool-17-*O*-[β-d-glucopyranosyl-(1→2)-{α-l-rhamnopyranosyl-(1→6)}]-β-d-glucopyranosid (Table 3, Figure 2).

Compound **4** with the molecular formula C_53_H_86_O_28_ was constructed from a basic carbon chain formed with 17 carbon atoms and two oligosaccharide chains (C-3 and C-17) like compounds **2** and **3** (Figure 2). The first sugar chain, attached at position C-3 of the diterpenic part was identical to that of compound **3**, the second one contained a 4,6 diglycosylated D-glucopyranosyl unit (Rha 1→6 Glc 6→1 Rha) identical to the Capsianoside VIII molecule, first identified by Marino et al. [37]. Therefore, compound **4** is 3-*O*-β-d-glucopyranosyl-(1→2)-β-d-glucopyranosyl-6-malonyl-17-hydroxy-6*E*,10*E*,14*Z*-(3*S*)-geranyllionalool-17-*O*-[α-l-rhamnopyranosyl-(1→4)-{α-l-rhamno-pyranosyl-(1→6)}]-β-d-glucopyranoside (Table 3, Figure 2).

## 3. Materials and Methods

### 3.1. Plant Material

The sweet pepper fruit cv. Ajfos was analyzed. Peppers were grown in the greenhouse of the University of Life Sciences in Lublin. The fruits were harvested at full maturity, washed, dried, and cut into cubes, with a thickness of 1 cm. The crushed material was frozen (−18 °C) and then lyophilized. Freeze drying was carried out for 72 h using a freeze dryer (Free Zone 12 lyophilizer, Labconco Corporation, Kansas City, MO, USA)) at −80 °C and 0.04 mbar.

### 3.2. Extract Preparation

The ground lyophilisate (0.5 g) was extracted with 80% ethanol (50 mL). The solvent extraction was accompanied by ultrasound (sonication for 10 min). The obtained extract was filtered using a vacuum pump, and its volume was reduced to 1 mL on a vacuum evaporator (Büchi, Flawil, Switzerland) at 40 °C. The concentrated extract was divided into 3 fractions with variable hydrophilicity by solid-phase extraction (SPE). The division was carried out on SPE-C18 columns (5 g, Sep-Pak, Waters, Milford, MA, USA). The hydrophilic fraction was eluted with water (F1), the intermediate lipophilic fraction with 40% methanol in water (F2), while the group of compounds with the highest hydrophobicity was eluted with 70% methanol in water (F3). The obtained fractions were lyophilized and subjected to spectrophotometric and chromatographic analyses after their dissolution in dimethyl sulfoxide (DMSO) to acquire solutions of known concentration (mg/mL).

### 3.3. Total Phenolic Compounds (TP)

The content of phenolic compounds was determined using the Folin-Ciocalteau reagent, according to the method described by Zheng and Wang [43]. The reaction mixture included the following reagents: 60 µL of the tested extract; 1.5 mL of Folin′s reagent eluted 1:10 in water; 1.2 mL sodium bicarbonate (75 g/L) and 0.54 mL distilled water. The reaction mixture prepared in this manner was stored at room temperature for 30 min, after which absorbance was measured at a wavelength of λ = 760 nm. The total content of phenolic compounds was expressed as a gallic acid equivalent [mg gallic acid/g lyophilized extract] based on the calibration curve for this compound prepared under the same assay conditions.

### 3.4. Total Flavonoids (TF)

The total flavonoid content was determined by the AlCl_3_ method [44]. The reaction mixture included the following reagents: 0.5 mL of the tested extract, 1.5 mL of ethanol (96%), 0.1 mL of AlCl_3_ (10%), 0.1 mL of sodium acetate (1 M), and 2.8 mL of distilled water. The reaction mixture was stored at room temperature for 40 min, and then absorbance was measured at a wavelength of λ = 415 nm. The sum of flavonoids was expressed as quercetin equivalent [mg quercetin/g lyophilized extract] based on a standard curve prepared for quercetin under the same conditions.

### 3.5. Total Dihydroxycinnamic Acids (TDHCA)

The content of dihydroxycinnamic acids was expressed as a chlorogenic acid equivalent. The reaction mixture contained 0.5 mL of the tested extract, 1 mL HCl (0.5 M), 1 mL of Arnov’s reagent (10 g NaNO_2_ + 10 g Na_2_MoO_4_ in 100 mL distilled water), 1 mL NaOH (2.125 M). Absorbance was measured 20 min after mixing the ingredients at a wavelength of λ = 525 nm [45].

### 3.6. Antiradical Activity

The prepared pepper extracts were also analyzed for antiradical activity, which was determined with the DPPH^•^ radical (1.1-diphenyl-2-picrylhydrazyl), according to the method provided by Conforti et al. [46] and relative to the cation radical ABTS^+•^ [2.2′-azino-bis (3-ethylbenzothiazoline-6-sulfonic acid)], in line with the methodology provided by Yang et al. [47]. In the system with DPPH^•^ radical, the reaction consisted of 100 mL of extract and 4 mL of 0.1 mM concentration of DPPH^•^ solution. Absorbance was measured at a wavelength of λ = 515 nm after 30 min mixing of the components. In the ABTS^+•^ radical system, a 7 mM ABTS^+•^ aqueous solution was prepared in a 2.45 mM potassium persulfate solution. ABTS^+•^ was prepared 12−16 h in advance and stored at room temperature in the dark. The initial solution was eluted until an absorbance value of 0.7 ± 0.2 was obtained. The reaction mixture contained a 3 mL ABTS^+•^ solution and a 20 mL test sample. Measurements were made at a wavelength of λ = 734 nm. Apart from the antiradical activities of the tested extracts, the activity of L-ascorbic acid and Trolox were analyzed as reference substances. The antiradical activity was expressed as the IC_50_ value (µg/mL), which refers to the extract concentration for which 50% inactivation of radicals present in the test sample was obtained.

### 3.7. Biological Activity

The cytotoxicity of subfractions with variable lipophilicity was assessed on two adherent tumor cell lines: human prostate cancer cells line (PC-3) and colorectal carcinoma cells (HTC116). The cells of PC-3 line carry a homozygous mutations in PTEN and TP53 genes. Furthermore, PC-3 cells are insensitive to androgens, glucocorticosteroids and epidermal growth hormone. The HCT116 cell line carries a mutation in codon 13 of the *RAS* proto-oncogene. The cytotoxicity of the fractions was evaluated in L929 mouse fibroblast cell line used in our experiment as a reference of normal cells. Fibroblast-like cells of L929 line obtained from mouse subcutaneous adipose tissue are recommended in the cytotoxicity testing. In vitro cytotoxicity studies were conducted in line with PN-EN ISO 10993-5:2009 norm. The HCT116, PC-3 and L929 cell lines were obtained from the American Type Culture Collection (ATCC, Rockville, MD, USA). The test has been optimized for the cell lines and extracts used in this experiment. PC-3 cell line was maintained in DMEM-F12 culture medium, while RPMI-1640 media was used for HCT116 cells. The normal cells were cultured in IMDM medium. All used media were supplemented with 10% fetal bovine serum and 1% penicillin/streptomycin. Cells were cultured in a humidified incubator allowing maintenance of 5% CO_2_ and 37 °C. The regular two-week passages were done using 0.025% trypsin/EDTA after the cells reached the state of 90% confluence. PC-3 and HTC116 cell lines were routinely checked for mycoplasma contamination.

MTT is a quantitative colorimetric method used to determine the state of cellular metabolism after treatment with the tested compounds. It is widely used to estimate the cytotoxic effect of chemicals on different types of cells [48]. MTT [3(4,5-dimethyl-2-thiazolyl)-2,5-diphenyl-2*H*-tetrazolium bromide] is a water-soluble tetrazolium salt, which is converted into an insoluble purple formazan crystals after cleavage of the tetrazolium ring by succinate dehydrogenase in mitochondria. The formazan product is impermeable to the cell membranes and therefore it accumulates in metabolically active cells. The assay was optimized for the cell lines and chemical compounds used in the experiments. The cancer cells and mouse fibroblasts (L929) were cultured for 24 h (37 °C, 5% CO_2_) on 96-well microplates with a density of 6−8 × 10^3^ cells/well. Then cells were incubated with tested extracts for the next 72 h under the same conditions. At the end of incubation, the medium was replaced with fresh MTT solution (5 mg/mL) for another 4 h. The contents of the wells were dissolved by adding 100 µl DMSO. The absorbance of the violet formazan solution was measured spectrophotometrically using a microplate reader (BioTek Instruments, Inc., Winooski, VT, USA) at 570 nm using DMSO as a blank. Results were analysed in the GraphPad Prism Version 7.0 (GraphPad Prism Software Inc., San Diego, CA, USA) software and presented as a IC_50_ values (median inhibitory concentration - concentration which is required for 50% inhibition of cells viability in comparison to negative control, which was accepted as 100%: % cell viability = {(Absorbance value of treated cells − Absorbance value of blank)/(Absorbance value of untreated cells − Absorbance value of blank)} × 100). 5-fluorouracil (a pyrimidine analogue used as an anticancer/antineoplastic agent to treat multiple solid tumors including colon, rectal, breast, gastric, pancreatic, ovarian, bladder and liver cancer) was applied as a positive control. Results were presented as mean ± SEM of replicates [49,50]. The dose –response curves of analyzed fractions are presented in Supplementary Material.

### 3.8. HPLC Analysis

The HPLC analysis was performed on a liquid chromatograph with a DAD detector (M2998), operated by the Empower-Pro software (Waters). The separation was carried out on an RP-18 column (Luna 3 µm, 4.6 mm x 150mm) in the following gradient system: A—100% ACN, B—redistilled water, C—100% MeOH: 0–15 min, 18–22% AC, 15–27 min, 22–25% AC, 27–35 min, 25–30% AC, 35–45 min, and 30–50% AC. The flow rate was 1 mL/min, and the sample volume dispensed was 20 µl. Detection of the obtained chromatographic separations was carried out at two wavelengths—λ = 280 nm and λ = 330 nm.

### 3.9. Isolation and Identification of Lipophilic Fraction Components

Isolation of lipophilic compounds from the 70% fraction was acquired from 500 g of freeze-dried pepper. The ground lyophilized pepper was extracted with 80% ethanol (1/100 *m*/*v*) in two stages with ultrasonic-bath-assisted extraction (10 min). The obtained pepper extracts were combined, filtered, and evaporated on a Büchi vacuum evaporator at 40 °C. The concentrated extract was divided into 3 fractions with variable hydrophilicity using solid phase extraction method (C18). Among other factors, the fraction of lipophilic compounds in 70% methanol/water was obtained and used for further analysis. This fraction was concentrated and applied to a preparative column (1.5 cm × 25 cm) filled with modified silica gel (40–60-µm, RP-18-LichroPrep, Merck, Darmstadt, Germany). The isolation process was performed through medium pressure chromatography on a Büchi apparatus, which included a fraction collector, a UV-Vis detector, and two medium pressure pumps. The compounds were eluted with a methanol-water mixture in a methanol concentration gradient of 0–100%. The separation of this fraction gave rise to 83 subfractions with a volume of 10 mL. The fractions were checked for the presence of phenolic compounds by thin-layer chromatography on cellulose coated plates (Merck). The developed chromatograms were analyzed under a UV lamp at two wavelengths—λ = 360 and λ = 254, and fractions with a similar profile were combined. Based on the obtained results, 20 subfractions were selected, which were purified by semipreparative HPLC. The purification was performed on a Knauer chromatograph (city, Germany) with a UV-Vis detector, on a Eurospher 100-C18 column (8 mm × 300 mm, 10 µm), at a flow rate of 2 mL/min, in a gradient acetonitrile (MeCN)−water solvent system, where the detector wavelength was 254 nm.

The purity of the isolated compounds was validated by ultra-high performance liquid chromatography (UPLC-MS) on a chromatograph equipped with a binary pump, autosampler, column changer, and PDA detector (Waters). The UPLC set was additionally connected with a mass spectrometer as a detector (ACQUITY TQD ESI MS, Waters, Milford, MA, USA). The obtained data were processed using MassLynx 4.1 and QuanLynx (Waters) software. The chromatographic separations were undertaken on the Atlantis T3 column (2.1 mm × 15 mm, 3 µM, Waters). The flow rate was 0.25 mL/min, temperature 50 °C. A two-gradient linear solvent system was used: A (Milli-Q water) and B (MeCN) both containing 0.1% HCOOH. The negative ionization mode was used for ESI-MS detection. Tandem mass spectrometry was chosen for the study because this system is widely used, owing to the stability of ion fragmentation. Optimal MS operating conditions were as follows: source temperature 140 °C and desolvation temperature 350 °C, atomizing gas flow (nitrogen), 80 L/h, and drying gas flow (nitrogen) 800 L/h.

Nuclear magnetic resonance analyses (NMR) were performed on a Bruker Advance 500 spectrometer (Bruker BioSpin GmbH, Rheinstetten, Germany) equipped with a 5 mm probe. ^1^H- NMR spectra (at 500 MHz) were measured at 298.15 K. Samples were dissolved in 250 µL CD_3_OD. Chemical shifts are given relative to tetramethylsilane (TMS) 0.00 ppm, using the residual solvent. Table 3 provides an interpretation of the data collected from NMR analyses.

### 3.10. Statistical Analyses

The results were obtained in three replications, and the data were expressed as the mean ± SD. The significance of differences between the means was determined with LSD one-way ANOVA test with 5% error probability. The dose–response curve was obtained by plotting the percentage of inhibition versus concentration. The inhibitory concentration of 50% (IC_50_) was calculated by linear and nonlinear regression analyses. Statistical comparisons were performed with the help of the Statgraphics Centurion software, version XVI (Statgraphics Technologies Inc. The Plains, VA, USA).

## 4. Conclusions

The scientifically established relationship between diet and human health has encouraged many countries to produce food enriched with natural antioxidants. The effectiveness of phenolic compounds as antioxidants extracted from plant materials differs, but this does not always depend on their quantity, and can also be related to the chemical structures of their components. Compounds with indirect hydrophilic and lipophilic properties included in pepper indicated high antiradical activity for DPPH^•^ and ABTS^+•^ radicals. Hence, they may play a significant therapeutic role in oxidative damage prevention. The anticancer activities were only related to the lipophilic fractions, which had low antiradical activity, which may be attributed to their glycosylation. The active compounds found in pepper fruits may be used as supportive factors in the prevention and, perhaps, treatment of civilization diseases, and lipophilic compounds may serve as potential therapeutic agents against cancer. New isolated and identified compounds contained in sweet peppers belong to the group of diterpenic derivatives, which exhibit anticancer properties, according to our research data. It may be presumed that individual compounds may display stronger activity against the examined cancer cells than their mixture in the analyzed fraction. However, their mutual symbiotic action cannot be excluded. Therefore, further experimental research is needed to determine the properties of single diterpenic derivatives, which may provide valuable information on their biological activity in vitro as well as in vivo.

## Figures and Tables

**Table 1:** Extraction yield, phenolic content, antioxidant, and biological activity of ethanolic extract (E) and water (F1), methanol-water 40% (F2), and 70% (F3) fractions obtained from sweet pepper fruit ^*^.

	Yield ^1^	Phenolics Content			Antiradical Activity ^5^		Biological Activity ^6^		
		TP ^2^	TF ^3^	TDHCA ^4^	DPPH ^•^	ABTS ^+•^	HCT116	PC-3	L929
Extract	6.98 ^a^ ± 0.05	83.25 ± 1.23	7.56 ^b^ ± 0.3	27.05 ^c^ ± 0.08	278 ^b^ ± 4.00	70.4 ^b^ ± 1.10	134 ^b^ ± 4.33	78^b^ ± 3.83	90 ^b^ ± 2.65
F1	5.82 ^b^ ± 0.07	155.64 ^c^ ± 3.70	7.32 ^b^ ± 0.01	26.08 ^c^ ± 0.13	284 ^b^ ± 3.75	44.6 ^c^ ± 1.01	158 ^a^ ± 3.82	60 ^c^ ± 3.42	64 ^c^ ± 2.70
F2	0.06 ^c^ ± 0.01	386.47 ^a^ ± 1.25	41.79 ^a^ ± 0.5	44.92 ^a^ ± 1.19	73 ^c^ ± 1.3	17.0 ^d^ ± 0.91	154 ^a^ ± 5.0	101 ^a^ ± 2.82	118 ^a^ ± 3.14
F3	0.11 ^c^ ± 0.03	172.09^b^ ± 0.41	6.49 ^c^ ± 0.3	33.56 ^b^ ± 0.04	570 ^a^ ± 8.1	111.7 ^a^ ± 1.20	160 ^a^ ± 3.86	51 ^d^ ± 4.42	94 ^b^ ± 3.20
Trolox	n.a.^8^	n.a.	n.a.	n.a.	5.5 ^d^ ± 0.08	3.4 ^e^ ± 0.03	n.a.	n.a.	n.a.
Ascorbic acid 5-fluorouracil ^7^	n.a. n.a.	n.a. n.a.	n.a. n.a.	n.a. n.a.	3.2 ^e^ ± 0.01 n.a.	0.2 ^f^ ± 0.03 n.a.	n.a. 32.87 ± 3.21	n.a. 23.3 ± 2.58	n.a. 7.5 ± 3.97

^*^ The values are expressed as the mean ±SD (n = 3). According to the LSD one way ANOVA test, means with a *p*-value less than 0.05 were considered to be statistically different. Taking this into account, the use of different letters in the same column indicates that there is a significant difference between results. ^1^ g/100g fresh pepper fruit; ^2^ Total phenolic content (mg gallic acid/g dry extract); ^3^ Total flavonoids (mg quercetin/g dry extract); ^4^ Total dihydroxycinnamic acids (mg chlorogenic acid/g dry extract); ^5,6^ IC_50_ (µg/mL); ^7^ 5-fluorouracil (µM)—positive control; ^8^ n.a.—not applicable

**Table 2:** Description of the Isolated Compounds.

No.	Chemical Name	T_r_	[M-H]^-^ *m*/*z*	Amount (mg)	Chemical Formula
**1**	Capsianoside IX	5.088	938	5.96	C_44_H_74_O_21_
**2**	new	9.664	1246	2.07	C_56_H_94_O_30_
**3**	new	10.296	1186	34.21	C_53_H_86_O_29_
**4**	new	17.398	1170	100	C_53_H_86_O_28_
**5**	Capsianoside VIII	21.832	1083	14.9	C_50_H_84_O_25_
**6**	Capsianoside I	27.124	659	1.47	C_32_H_52_O_14_
**7**	Capsianoside IV	36.531	805	1.6	C_38_H_62_O_18_
**8**	Capsianoside III	38.743	1099	13.91	C_50_H_84_O_26_

**Table 3:** ^1^H- and ^13^C-NMR Analysis of compounds **2–4**.

	2		3		4	
Position	δ_H_^a^	δ_C_ CD_3_OD (25 °C)	δ_H_^a^	δ_C_ CD_3_OD (25 °C)	δ_H_^a^	δ_C_ CD_3_OD (25 °C)
1	5.22 dd(10.5. 1.4) 5.23 dd(17.8. 1.4)	116.1	5.23 dd(11.0; 1.3) 5.25 dd(17.8. 1.4)	116.3	5.23 dd(11.0; 1.4) 5.25 dd(17.8. 1.4)	116.2
2	6.13 dd(18.1. 10.7)	144.4	6.08 dd(17.7; 11.0)	144.1	6.8 dd(17.7; 11.0)	144.1
3	-	82.1	-	82.2	-	82.2
4	1.60 m	43.1	1.59	43.0	1.60 m	43.0
5	2.06 m	23.6	2.05 o	23.5	2.05 o	23.5
6	5.13 m	125.8	5.13 o	125.7	5.13 o	125.7
7		136		136		136
8	1.99 m	40.8	2.00 o	40.8	1.99 o	40.8
9	2.09 m	27.7	2.09 o	27.7	2.09 o	27.7
10	5.13 m	125.9	5.13 o	126.0	5.12 o	125.9
11	-	135.5	-	135.4	-	135.5
12	2.01 t(4.5)	40.9	2.02 t(7.1)	40.9	2.01 t(7.4)	40.9
13	2.17 m(7.30)	27.3	2.17 m(7.4)	27.2	2.17 m(6.9)	27.3
14	5.40 dd(8.0 6.4)	131.3	5.39 dd(8.0; 6.2)	131.3	5.40 td(7.3; 1.7)	131.3
15	-	132.4	-	132.4	-	132.4
16	1.77 d(1.4)	21.9	1.79 d(1.5)	22.0	1.77 d(1.5)	21.9
17	4.33 d(11.5) 4.13 d(11.6)	67.7	4.30 d(11.4) 4.21 d(11.6)	68.2	4.33 d(11.5) 4.13 d(11.5)	67.7
18	1.61 s	16.3	1.61 s	16.3	1.61 s	16.3
19	1.61 s	16.2	1.61 s	16.3	1.61 s	16.3
20	1.39 s	23.4	1.36 s	23.5	1.39 s	23.5
1′			-	168.7	-	168.7
2′			3.37 o	41.9	3.37 o	41.9
3′			-	170.1	-	170.1
ß Glc (1) ^1^*J*_CH_ *=* 158						
1	4.47 d(7.7)	98.4	4.48 d(7.6)	98.2	4.48 d(7.7)	98.2
2	3.44 dd(9.3. 7.7)	83.2	3.46 dd(9.9; 7.8)	82.9	3.46 dd(9.4; 7.8)	82.9
3	3.51 t(9.0)	78.1	3.52 t(8.4)	77.9	3.52 t(8.9)	77.9
4	3.31 t(9.3)	71.6	3.30 t(9.1)	71.7	3.29 dd(9.8; 8.8)	71.7
5	3.16 ddd(9.0. 5.5. 2.4.)	77.5	3.41ddd(9.5; 5.5; 2.0)	74.8	3.41 ddd(9.2; 6.8; 2.2)	74.8
6	3.81 dd(12.0 2.2) 3.64 dd(11.9. 5.6)	62.7	4.42 dd(11.8; 2.0) 4.22 dd(12.0; 5.5)	65.6	4.43 dd(11.8; 2.1) 4.22 dd(12.5; 5.8)	65.6
ß Glc (2) ^1^*J*_CH_ *=* 160						
1	4.2 d(7.9)	102.1	4.36 d(7.7)	101.0	4.21 d(7.9)	102.1
2	3.21 dd(9.1. 7.8)	75.3	3.48 dd(9.3; 7.5)	81.9	3.22 dd(9.1; 7.9)	75.2
3	3.42 t(9.0)	76.7	3.56 t(9.0)	78.1	3.44 t(9.0)	76.7
4	3.58 t(9.4)	78.9	3.36 o	71.3	3.58 t(9.3)	79.2
5	3.37 ddd(9.7; 3.8; 2.0)	75.4	3.36 o	76.6	3.36 ddd(9.7; 3.9; 1.7)	75.4
6	3.93 dd(11.0; 1.8) 3.61 dd(11.0; 3.9)	66.8	3.97 d(11.0) 3.63 dd(11.0; 4.0)	67.6	3.93 dd(11.4; 1.4) 3.62 dd(11.1; 3.8)	66.8
ß Glc (3) ^1^*J*_CH_ *=* 158						
1	4.56 dd(7.7)	105.9	4.56 d(7.7)	105.8	4.56 d(7.7)	105.8
2	3.25 t(8.4)	76.6	3.24 t(8.9)	76.6	3.24 dd(9.1; 7.08)	76.6
3	3.39 t (9.1)	77.7	3.37 t (8.7)	77.7	3.38 t(9.0)	77.7
4	3.35 t(8.6)	71.4	3.34 t(8.5)	71.4	3.34 t(8.9)	71.4
5	3.26 ddd(9.5;4.9.; 2.3)	78.3	3.26 o	78.3	3.26 ddd (9.3; 5.0; 2.4)	78.3
6	3.83 dd(11.6; 2.4) 3.71 dd(11.9; 5.0)	62.7	3.82 d(11.4) 3.70 dd(11.9; 4.8)	62.7	3.82 dd(11.7; 2.4) 3.70 dd(11.9; 5.0)	62.7
ß Glc (4) ^1^*J*_CH_ *=* 160						
1	4.58 d(7.8)	105.6	4.63 d(7.8)	104.8		
2	3.21 dd(9.1; 7.8)	76.0	3.24 dd(9.2; 7.8)	75.9		
3	3.37 t(8.8)	78.2	3.37 t(8.8)	77.6		
4	3.31 dd(9.5; 8.2)	71.5	3.30 t(9.7)(9.5; 8.2)	71.5		
5	3.28 ddd(9.3; 5.2; 2.1)	78	3.26 o	78.2		
6	3.85 dd(11.9; 2.3) 3.69 dd(11.8; 5.0)	62.7	3.83 dd(12.0; 2.3) 3.67 dd(11.9; 5.0)	62.8		
α Rha (1) ^1^*J*_CH_ = 168						
1	4.71 d(1.7)	101.6	4.75 d(1.7)	102.1	4.71 d(1.7)	101.6
2	3.85 dd(3.6; 1.70	72.2	3.85 dd(3.5; 1.7)	72.2	3.85 dd(3.5; 1.5)	72.2
3	3.69 dd(9.5; 3.3)	72.3	3.66 dd(9.5; 3.3)	72.4	3.69 dd(9.5; 3.3)	72.3
4	3.37 t(9.5)	74.0	3.37 t(9.5)	74.0	3.37 t(9.5)	74.0
5	3.72 dq(9.5; 6.2)	69.8	3.67 dq(9.4; 6.2)	69.7	3.72 dq(9.7; 6.2)	69.8
6	1.27 d (6.2)	18.2	1.27 d(6.2)	18.1	1.27 d(6.2)	18.2
α Rha (2) ^1^*J*_CH_ = 168						
1	4.82 d(1.7)	102.3			4.82 d(1.8)	102.6
2	3.85 dd(3.3; 1.8)	72.3			3.83 dd(3.4; 1.8)	72.5
3	3.88 dd(9.1; 3.7)	72.3			3.64 dd(9.4; 3.3)	72.2
4	3.63 t(9.3)	83.3			3.40 t(9.5)	73.8
5	4.09 dq(9.6; 6.1)	69.2			3.99 dq(9.6; 6.2)	70.6
6	1.34 d(6.2)	18			1.26 d (6.2)	17.8

## Supplementary Materials

Figure S1: Thye dose-response curves of biological activity of tested fractions.
